# Tissue-specific responses of oxidative stress biomarkers and antioxidant defenses in rainbow trout *Oncorhynchus mykiss* during a vaccination against furunculosis

**DOI:** 10.1007/s10695-014-9924-9

**Published:** 2014-03-06

**Authors:** Halyna Tkachenko, Natalia Kurhaluk, Joanna Grudniewska, Anastasiia Andriichuk

**Affiliations:** 1 Department of Zoology and Animal Physiology, Institute of Biology and Environmental Protection, Pomeranian University, Slupsk, Poland; 2 Department of Salmonid Research, Inland Fisheries Institute, Rutki, 83-330 Żukowo, Poland

**Keywords:** Lipid peroxidation, Oxidatively modified proteins, Antioxidant enzymes, Muscle, Gills, Liver, Brain, Rainbow trout *Oncorhynchus mykiss*

## Abstract

The present study was conducted to evaluate the effects of vaccination against furunculosis on responses of oxidative stress and antioxidant defenses in rainbow trout *Oncorhynchus mykiss* muscle, gills, liver, and brain tissues. The oxidative stress markers (malondialdehyde and carbonyl derivatives of protein oxidative destruction levels), antioxidant defenses (superoxide dismutase, catalase, glutathione reductase, and glutathione peroxidase), and total antioxidant capacity in different tissues of rainbow trout were measured. Our data showed that exposure of trout to vaccine against furunculosis produced changes (either increase or decrease) in oxidative stress and antioxidant enzymes responses, and these responses showed marked organ differences, associated with tissue patterns. Our study demonstrated that vaccinated trout showed alteration in antioxidant defenses and oxidative stress responses, with higher severity in the liver, compared with other tissues. Our data also suggest that vaccination against furunculosis induced lipid peroxidation in gill and liver tissues. However, muscle and brain tissue are capable of restoring its pro- and antioxidant balance after vaccination.

## Introduction

Salmonids are an important species for pond aquaculture and extensive open water fisheries in several European countries. In Poland, the intensification of salmonids production is occurring, especially in water recirculation systems. Rapid growth and disease resistance are the most important concerns in the present aquaculture industry (Andrews et al. 2011).

The most important bacterial fish diseases in European freshwater aquaculture are the rainbow trout fry syndrome (*Flavobacterium psychrophilum*) and enteric redmouth disease (*Yersinia ruckeri*) that are widespread and cause serious epizootics, while furunculosis (*Aeromonas salmonicida*) is endemic, only giving overt disease under extremely stressing conditions (Larsen and Pedersen 1997). Salmonids are vulnerable to furunculosis, a disease caused by the Gram-negative bacterium *Aeromonas salmonicida* and *Aeromonas hydrophila* (Vanya Ewart et al. 2008; Swain et al. 2010). Infections with *A. salmonicida* and *A. hydrophila* are probably the most important disease problems in European aquaculture as they are widespread and cause disease both in fresh water and sea water (Press and Lillehaug 1995). The term furunculosis is derived from the characteristic furuncles in muscles, which are common during a chronic course of the disease. Otherwise, the dominant pathological findings are a swollen, dark spleen, and petechial hemorrhages in internal organs (Press and Lillehaug 1995).

Increased incidence of infectious diseases connected with *Aeromonas* infection has traditionally been treated with antibiotics, chemotherapeutics, and vaccines (Harikrishnan et al. 2010). The use of immunostimulants in fish for the modulation of nonspecific defense mechanisms and protection against infectious diseases is a promising new development (Siwicki et al. 1994, 1998; Bricknell and Dalmo 2005). So vaccination is the most promising concept to control disease (Swain et al. 2010). Vaccination against furunculosis reduces mortality of salmonids but fails to eradicate infection. Efficiency of vaccination depends largely on the ability of host to neutralize the negative impacts of immune responses combined with efficient clearance and prevention of tissue damages (Skugor et al. 2009). Since the introduction of successful oil-adjuvanted vaccines in the early 1990s, a number of studies have been published on the protective as well as adverse effects of these vaccines (Rømer Villumsen et al. 2012).

Different kinds of vaccines have been investigated against *A. hydrophila* including whole cell, outer membrane proteins, extra-cellular proteins, lipopolysaccharides, and biofilms-attenuated vaccines (Loghothetis and Austin 1994; Karunasagar et al. 1997; Siwicki et al. 1998; Rahman and Kawai 2000; Azad et al. 2000; Vivas et al. 2004). While each medicine probably are effective in the treatment of a particular disease, problems arise with the development of possible pathological side effects of immunization in fishes, as well as the emergence of antibiotic resistant pathogenic strains. For optimal protection of salmonids in seawater, vaccination should be carried out sometime before sea transfer, in order to give immunity sufficient time to develop, and to avoid handling stress during smoltification. On the other hand, however, vaccination should not be carried out too early, as the degree of immunity declines with time (Sommerset et al. 2005). Water temperature is an important factor when deciding when to vaccinate (Lillehaug 1997). Results of Skugor et al. (2009) suggest that outcomes of vaccination depend largely on the ability of host to prevent the negative impacts of immune responses and to repair damages. This can be illustrated with the inductions of protease inhibitors, negative regulators of complement, and genes involved in metabolism of lipids and xenobiotics and growth factors. There was no strong association between survival and most anti-bacterial responses though vaccinated fish with high resistance to furunculosis (Skugor et al. 2009).

Despite the importance and success of vaccination, little is known about the mechanisms of oxidative stress and antioxidant defenses in fish during vaccination. In the present study, we determined the influence of vaccination against furunculosis on tissue-specific responses of oxidative stress biomarkers and antioxidant defense in rainbow trout, *Oncorhynchus mykiss*. In the present work, a comparative study is made concerning the oxidative stress biomarkers (lipid peroxidation and oxidatively modified protein levels), as well as activities of the antioxidant enzymes (superoxide dismutase, catalase, glutathione reductase, and glutathione peroxidase) and total antioxidant capacity in white muscle, gills, liver, and brain tissues of the rainbow trout *O. mykiss* treated by vaccine against furunculosis.

## Materials and methods

### Experimental animals

Clinically healthy rainbow trout (*Oncorhynchus mykiss* Walbaum) with a mean body mass of 135.5 ± 1.5 g were used in the experiments. The study was carried out in a Department of Salmonid Research, Inland Fisheries Institute near the village of Żukowo (Poland). The experiments were conducted with the current laws in Poland, according to the guidelines of Local Ethical Commission.

Experiments were performed at a water temperature of 14.5 ± 0.5 °C, and the pH was 7.5. The dissolved oxygen level was about 12 ppm with additional oxygen supply with a water flow of 25 L/min and a photoperiod of 7 h per day. The fish were fed with commercial pelleted diet. All enzymatic assays were carried out at Department of Animal Physiology, Institute of Biology and Environmental Protection, Pomeranian University (Słupsk, Poland).

### Experimental design

The fish were divided into two groups: (1) unhandled control, (2) vaccinated by vaccine against furunculosis. Fish were held in 250-L square tanks (70–75 fish per tank) with the same conditions. Before vaccination, the fish were anaesthetized by Propiscin solution (Siwicki et al. 2002). The vaccine against furunculosis is a vaccine containing an inactivated strain of *A. salmonicida* and *A. hydrophila* in concentration 1 × 10^10^ colony-forming units (CFU). The vaccine was produced in Department of Epizootology, Faculty of Veterinary Medicine, University of Warmia and Mazury (Olsztyn, Poland). Immersion contained 1 liter of vaccine per 10 L of water. It was prepared immediately prior to vaccination. Immersion lasted from 60 to 120 s. The fish were kept for 30 days at 14.5 °C after vaccination at a water temperature of 14.5 ± 0.5 °C and the pH 7.5. In our study, 15 rainbow trout from unhandled control and 15 vaccinated trout were used.

### Sampling

The animals were quickly captured and killed on 31 days post vaccination (*n* = 15 in each group). White muscle, gills, liver, and brain tissues were removed in situ. Tissue samples were homogenized in ice-cold buffer (100 mM Tris–HCl, pH 7.2) using a glass homogenizer immersed in an ice-water bath to a yield a 10 % homogenate. Homogenates were centrifuged at 3,000*g* for 15 min at 4 °C. After centrifugation, the supernatant was collected and frozen at −20 °C until analyzed. Protein contents were determined using the method of Bradford (1976) with bovine serum albumin as a standard. Absorbance was recorded at 595 nm. All enzymatic assays were carried out at 22 ± 0.5 °C using a Specol 11 spectrophotometer (Carl Zeiss Jena, Germany) in duplicate. The enzymatic reactions were started by the addition of the tissue supernatant. The specific assay conditions were as follows.

### Oxidative stress biomarkers assay

#### Assay of thiobarbituric acid reactive substances (TBARS) levels

An aliquot of the homogenate was used to determine the lipid peroxidation status of the sample by measuring the concentration of thiobarbituric acid-reacting substances (TBARS), according to the method of Kamyshnikov (2004). Reaction mixture contains sample homogenate (2.1 mL, 10 % w/v) in Tris–HCl buffer (100 mM, pH 7.2), 2-thiobarbituric acid (TBA; 0.8 %, 1.0 mL), and trichloracetic acid (TCA; 20 %, 1.0 mL). The total volume was kept in a water bath at 100 °C for 10 min. After cooling, mixture was centrifuged at 3,000*g* for 10 min. The absorbance of the supernatant was measured at 540 nm. TBARS values were reported as nmoles malondialdehyde (MDA) per mg protein.

#### Assay of carbonyl groups of oxidatively modified protein levels

Carbonyl groups were measured as an indication of oxidative damage to proteins according to the method of Levine et al. (1990) in modification of Dubinina et al. (1995). Samples were incubated at room temperature for 1 h with 10 mM 2,4-dinitrophenylhydrazine (DNTP) in 2 M HCl. Blanks were run without DNTP. Afterward, proteins were precipitated with TCA and centrifuged for 20 min at 3,000*g*. The protein pellet was washed three times with ethanol/ethylacetate (1:1) and incubated at 37 °C until complete resuspension. The carbonyl content could be measured spectrophotometrically at 370 nm (aldehyde derivates, OMP_370_) and at 430 nm (ketonic derivates, OMP_430_) (molar extinction coefficient 22,000 M^−1^ cm^−1^) and expressed as nmol per mg protein.

#### Assay of superoxide dismutase activity

Superoxide dismutase (SOD, E.C. 1.15.1.1) activity in supernatant was determined according to the Kostiuk et al. (1990). SOD activity was assessed by its ability to dismutate superoxide produced during quercetin auto-oxidation in an alkaline medium (pH 10.0). Briefly, 1.0 mL of C reagent was mixed with 0.1 mL homogenate (1:1,000). C reagent was made *ex tempore* (mixture of equal volumes of 0.1 M K, Na phosphate buffer, pH 7.8, and 0.08 M EDTA); pH of C reagent was adjusted to 10.0 by adding tetramethylenediamine. Distilled water (0.1 mL) was added to blank instead of homogenate. The total volume was then made up to 2.4 mL with distilled water. The reaction was initiated by adding 0.1 mL of quercetin (1.4 μM). Absorbance at 406 nm was measured immediately and after 20 min. Activity is expressed in units of SOD per mg of tissue protein.

#### Assay of catalase activity

Catalase (CAT; EC 1.11.1.6) activity was determined by measuring the decrease in H_2_O_2_ concentration at 410 nm according to Koroliuk et al. (1988). The rate of decrease in H_2_O_2_ content is directly proportional to the CAT activity in the sample. Assays were performed in a reaction mixture containing 2 mL of 0.03 % H_2_O_2_ solution and 0.1 mL of tissue homogenate. The duration of this reaction was 10 min at room temperature. The reaction was terminated by rapid adding of 1.0 mL of 4 % ammonium molybdate dissolved in 12.5 mM H_2_SO_4_ and 1 mL of 125 mM H_2_SO_4_. All samples were centrifuged at 3,000*g* for 10 min. One unit of CAT activity was defined as the decrease of 1 μmol in H_2_O_2_ per minute. CAT activities were expressed as one unit per milligram protein.

#### Assay of glutathione reductase activity

Glutathione reductase (GR, EC 1.6.4.2) activity was assayed as described by Glatzle et al. (1974) with some modifications. The enzymatic activity was assayed spectrophotometrically by measuring NADPH consumption. In the presence of GSSG and NADPH, GR reduces GSSG and oxidizes NADPH, resulting in a decrease in absorbance at 340 nm. Quantification was based on the molar extinction coefficient of 6.22 mM^−1^ cm^−1^ of NADPH. One unit of GR was defined as the amount of enzyme that reduced 1 μmol of NADPH with GSSG per minute. The GR activities were expressed as one unit per mg protein.

#### Assay of glutathione peroxidase activity

Glutathione peroxidase (GPx, EC 1.11.1.9) activities were determined on the detection of nonenzymatic utilization of GSH as the reacting substrate at an absorbance of 412 nm after incubation with 5,5-dithiobis-2-nitrobenzoic acid (DTNB) according to the method of Moin (1986). Briefly, reaction mixture contained 0.8 mL of 0.1 M Tris–HCl buffer (pH 8.9) with 12 mM sodium azide, 6 mM EDTA, 0.2 mL of homogenate, 0.1 mL of 4.8 mM glutathione, and 0.1 mL of 20 mM t-butyl hydroperoxide. The contents were incubated at 37 °C for 10 min. The reaction was arrested by 0.2 mL of 20 % TCA and centrifuged. Supernatant was assayed for glutathione content using Ellman’s reagent (39.6 mg of DTNB in 10 mL of 1 % sodium citrate). A unit of enzyme activity is defined as the amount of enzyme catalyzing the formation of 1 μM of GSH per min, and the activity of GPx was calculated based on tissue protein concentration.

#### Assay of total antioxidant capacity (TAC)

The TAC level was estimated spectrophotometrically at 532 nm following the method with Tween 80 oxidation (Galaktionova et al. 1998). Briefly, 0.2 mL of tissue homogenate was added to 2 mL of 1 % Tween 80. Blank assay instead of sample included 0.1 mL of distilled water. The mixture was incubated during 48 h at 37 °C. After cooling, 1 mL of 40 % TCA was added. The mixture was centrifuged at 3,000*g* for 10 min. After centrifugation, 2 mL of supernatant and 2 mL of 0.25 % TBA reagent were mixed. The mixture was heated in boiling water bath at 100 °C for 15 min. The absorbance of the obtained solution was measured at 532 nm and was compared with the blank. TAC level was expressed in %.

### Statistical analysis

Data were presented as the mean ± SEM and were checked for assumptions of normality using the Kolmogorov–Smirnov one-sample test and Lilliefors test (*p* > 0.05). Homogeneity of variance was checked using the Levene’s test. One-way analysis of variance (ANOVA) was used to check for differences between the control and vaccinated groups. Significance of differences in the lipid peroxidation level, level of carbonyl derivatives of amino acids reaction, and antioxidant enzymes activities was examined using Mann–Whitney *U* test according to Zar (1999). Differences were considered significant at *p* < 0.05. All statistical analysis was performed by STATISTICA 8.0 software (StatSoft, Poland).

## Results

**Fig. 1:**
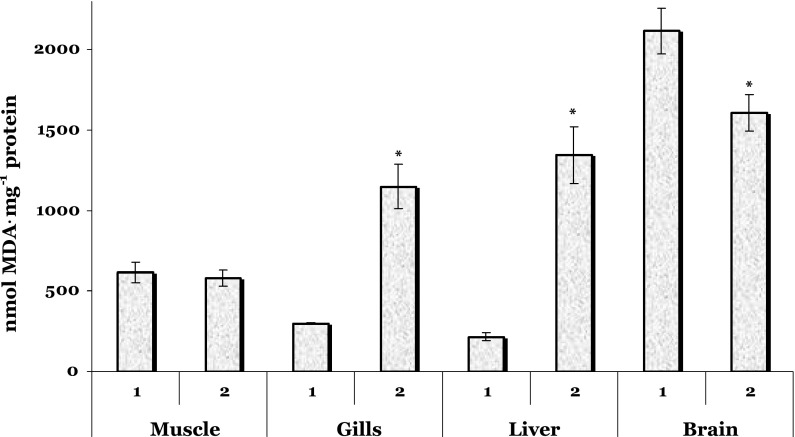
The level of lipid peroxidation (nmol MDA per mg protein) in the muscle, gills, liver, and brain of the trout treated by vaccine against furunculosis. *1* unhandled controls, *2* trout treated by vaccine against furunculosis. Data are represented as mean ± SEM (*n* = 15). *Asterisk* Significant difference was shown as *p* < 0.05 when the vaccinated group and unhandled group values were compared

The level of lipid peroxidation in the muscle tissue of trout treated by vaccine did not significantly differ from that in the controls (Fig. 1). The gill and hepar tissues of vaccinated trout demonstrated significantly higher TBARS levels (3.82- and 6.25-fold, *p* = 0.000, respectively) compared with unhandled control fish (Fig. 1). Vaccination caused a significant decrease in the TBARS level of the brain tissue by 24 % (*p* = 0.017).

**Fig. 2:**
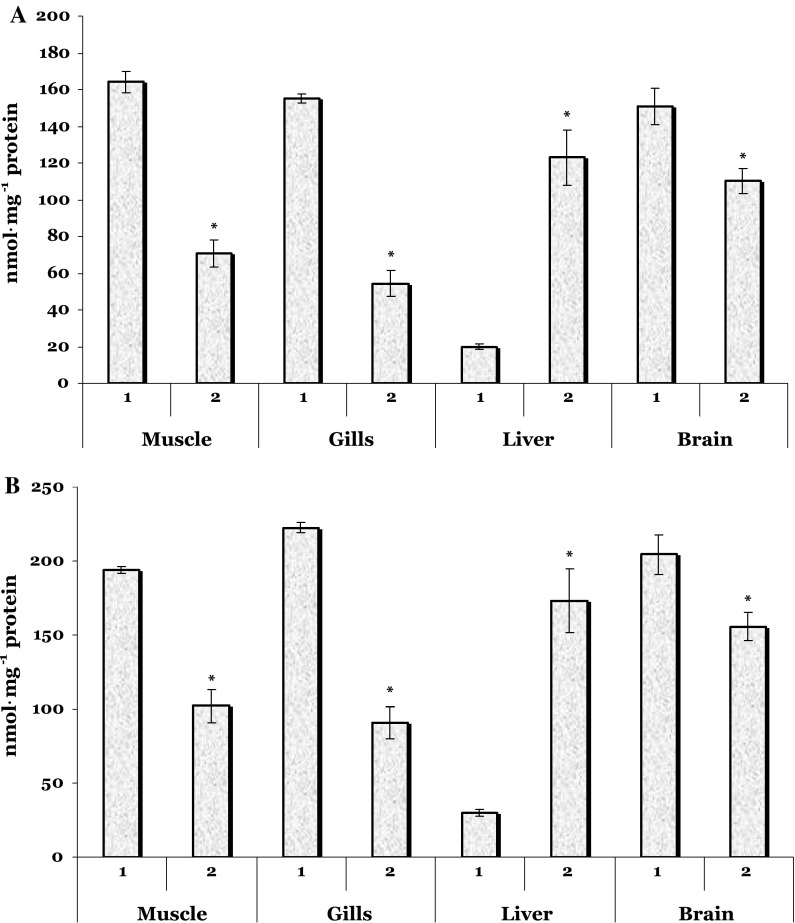
Aldehyde (**a**) and ketonic derivates (**b**) of carbonyl content in the muscle, gills, liver, and brain of the trout vaccinated against furunculosis. *1* unhandled controls, *2* trout treated by vaccine against furunculosis. Data are represented as mean ± SEM (*n* = 15). *Asterisk* Significant difference was shown as *p* < 0.05 when the vaccinated group and unhandled group values were compared

Aldehyde and ketonic derivates of carbonyl content in the trout vaccinated against furunculosis were significantly reduced in the muscle (by 57 % and by 47.5 %, *p* = 0.000, respectively), gills (by 65 % and by 59 %, *p* = 0.000, respectively), and brain tissue (by 27 %, *p* = 0.008 and by 24 %, *p* = 0.006, respectively) compared with the level in the control (Fig. 2). In contrast, significantly increased carbonyl content was measured in the liver of fish exposed to vaccine in comparison with the control values (6.2- and 5.8-fold for aldehyde and ketonic derivates, respectively) (Fig. 2).

The activities of antioxidant enzymes in the muscle, gill, liver, and brain tissues in response to vaccine treatment are presented in Table 1. No significant change in SOD activity in the gills and brain tissue was observed for vaccinated group compared with the control activities level. Muscle and liver SOD activity was significantly higher than that in the control (3.5- and 2.1-fold, respectively). CAT, GR, and GPx activities in the muscle and gill were also significantly inhibited in vaccinated fish. In contrary, hepatic CAT, GR, and GPx activities were significantly increased compared with the control activities (Table 1).

**Table 1:** Enzymatic antioxidant defenses in the muscle, gills, liver, and brain tissue of the trout vaccinated against furunculosis

Antioxidant enzymes	Muscle		Liver		Brain		Gills	
	Unhandled control	Vaccinated group	Unhandled control	Vaccinated group	Unhandled control	Vaccinated group	Unhandled control	Vaccinated group
SOD, U mg^−1^ protein	292.3 ± 6.34	1,036.75 ± 138.11*	327.95 ± 14.87	695.34 ± 74.77*	749.89 ± 48.18	780.12 ± 156.72	397.88 ± 27.54	453.64 ± 48.53
CAT, μmol min^−1^ mg^−1^ protein	29.46 ± 5.24	19.5 ± 5.51*	18.26 ± 1.24	117.22 ± 14.03*	119.67 ± 10.21	95.74 ± 6.91	105.5 ± 4.21	48.15 ± 6.17*
GR, μmol min^−1^ mg^−1^ protein	1.49 ± 0.09	1.39 ± 0.22*	0.26 ± 0.026	2.32 ± 0.31*	1.75 ± 0.20	1.96 ± 0.21	1.92 ± 0.09	0.98 ± 0.17*
GPx, μmol min^−1^ mg^−1^ protein	1,134.92 ± 173.18	425.48 ± 57.67*	175.79 ± 29.80	1,095.94 ± 179.76*	1,104.15 ± 183.13	945.65 ± 149.80	1,382.51 ± 171.49	641.98 ± 99.43*

Data are represented as mean ± SEM (*n* = 15)

* Significant difference was shown as *p* < 0.05 when the vaccinated group and unhandled group values were compared

**Fig. 3:**
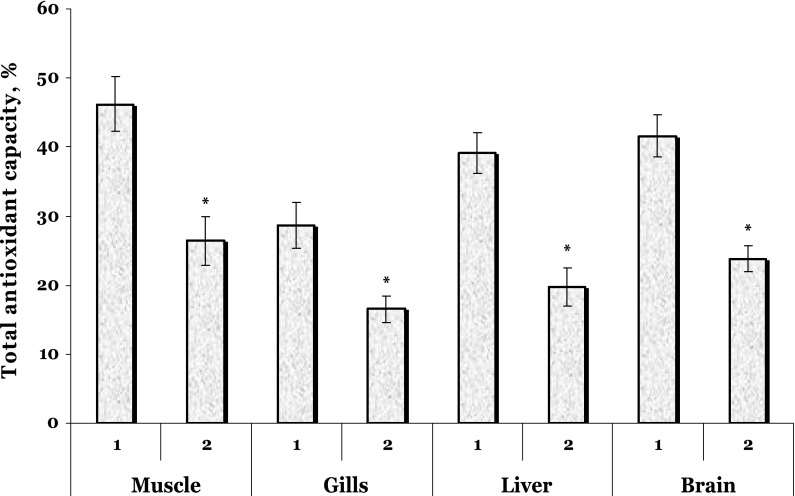
The total antioxidant capacity in the muscle, gills, liver, and brain of the trout treated by vaccine against furunculosis. *1* unhandled controls, *2* trout treated by vaccine against furunculosis. Data are represented as mean ± SEM (*n* = 15). *Asterisk* Significant difference was shown as *p* < 0.05 when the vaccinated group and unhandled group values were compared

The total antioxidant capacity (Fig. 3) was significantly decreased in all tissues compared with those in the control (in muscle by 43 %, *p* = 0.002, in gills by 42 %, *p* = 0.012, in liver by 50 %, *p* = 0.006, and in brain by 43 %, *p* = 0.000).

## Discussion

The study shows a post-treatment change in oxidative stress profile in the muscle, gills, liver, and brain of rainbow trout treated by vaccine against furunculosis. Our study showed that treatment with vaccine resulted in significant reduction in lipid peroxidation biomarker level in brain tissue. The decrease in aldehyde and ketonic derivates of carbonyl content in gills, brain and muscle tissues was observed. However, the post-treatment levels of oxidative stress biomarkers in the liver tissue showed increase after vaccination.

Certain conditions (such as disease, exposure to toxins, immunization, aging, and exercise) can increase the rate of oxidative damage, a condition called oxidative stress (Pryor 1986). Oxidative stress occurs when the critical balance between oxidants and antioxidants is disrupted due to the depletion of antioxidants or excessive accumulation of the reactive oxygen species (ROS), or both, which may lead to a series of biochemical and physiological changes, thus, altering normal body homeostasis and tissue injury (Halliwell 1994). Despite the potential danger of the ROS, cells have a variety of defense mechanisms to neutralize the harmful effects of free radicals (Ural 2013).

The present study established that the gills and liver of fish vaccinated against furunculosis have higher level of lipid peroxidation. Skin, gills, and gut are the mucosal tissues associated with the immune system of fish (Kirron 2012). Gills are multifunctional—primarily a respiratory organ, they are also involved in the immune defense through the mucosa-associated lymphoid tissues that harbor macrophages, neutrophils, lymphocytes, and mast cells/eosinophilic granulocytes (Pratap and Wendelaar Bonga 1993; Reite and Evensen 2006). Since the gills are in continuous close contact with the aquatic environment, perhaps the higher level of TBARS in gills is due to their function. Fish gills are particularly sensitive to water quality, constituting the first target of pollutants, due to their anatomic location, direct contact with the water and quick absorption (Pereira et al. 2013). In our study, higher level of TBARS in the gills of vaccinated fish apparently is caused by close contact of gills tissue with the aquatic environment. They are also involved in the immune defense through the mucosa-associated lymphoid tissues that harbor macrophages, neutrophils, lymphocytes, and mast cells/eosinophilic granulocytes (Kirron 2012). It well-known phagocytic cells, i.e., neutrophils, monocytes, and macrophages, generate ROS during the respiratory burst by membrane-bound NADPH oxidase and play an important role in defense against microorganisms and various exogenous compounds (Rossi 1986; Pietarinen-Runtti et al. 2000).

Liver is a metabolically active organ with a powerful antioxidant potential. Therefore, TBARS level was higher in the liver than in the other tissues. Our study is in agreement with the data of other researches (Velisek et al. 2011). The significant increase of oxidative stress biomarkers level in liver and gills of vaccinated fish may indicate susceptibility of lipid molecules to ROS and demonstrate the extent of oxidative damage under the influence vaccination against furunculosis.

Skugor et al. (2009) used multiple gene expression profiling to outline the mechanisms that determine success of vaccine protection against furunculosis in Atlantic salmon and to search for the correlates of protection. Several genes with known immune functions showed higher expression levels in liver of salmon, including the phosphotyrosine-independent ligand for lymphocyte-specific protein tyrosine kinase Lck SH2 or nucleoporin p62 that regulates activation of nuclear factor kappa light-chain enhancer of activated B cells (NF-kB) by tumor necrosis factor α (TNFα) (Skugor et al. 2009). Up-regulation of NF-kB and activator protein AP-1 by pathogens and cytokines induces mass production of immune mediators and effector proteins. NF-kB and Jun proteins respond to various cell-damaging factors, including free radicals and other genotoxic agents that can cause apoptosis, growth arrest, altered DNA repair, or altered differentiation. NF-kB can also activate protection against oxidative and cellular stress by providing anti-apoptotic and proliferation-promoting signals. A suite of chaperones and protein adaptors of different types (heat shock proteins, 14–3–3 proteins, glucose regulated proteins, DnaJ, and cyclophilins) were expressed at higher level in fish with low resistance to furunculosis, and this could be evidence of cellular stress (Skugor et al. 2009). Genes for proteins involved in regulation of redox status and protection against ROS had higher expression levels in vaccinated fish with high resistance to furunculosis (Skugor et al. 2009).

The complement system is the only group of immune genes that showed strong association with survival and can be considered as candidate markers of vaccine protection against furunculosis. The role of complement in antibody-mediated defense against *A. salmonicida* was demonstrated in rainbow trout. The complement system is the major link between the effector anti-bacterial mechanisms of adaptive and innate arms of immunity. The phagocytes of fish play an important role in innate host defense against bacterial infection and participate in various immunoregulatory processes (Skugor et al. 2009). Nikoskelainen et al. (2005) investigated the effects of various opsonins in the ingestion and adhesion processes by examining respiratory burst activity in blood and head kidney fish phagocytes. Combination of specific IgM and complement accelerated ingestion of target bacteria and ingestion-activated respiratory burst in phagocytes (Nikoskelainen et al. 2005). The antibody-mediated opsonophagocytosis was determined as the respiratory burst activity of blood monocytes and granulocytes against bacterial antigens (Nikoskelainen et al. 2007). ROS production is an important effector mechanism mediating intracellular killing of microbes by phagocytes. On the other side, inappropriate or untimely ROS production can lead to tissue damage (Steevels et al. 2013).

The oxidative modification of protein (OMP) could be used as molecular markers of oxidative stress (Levine et al. 1990). Bagnyukova et al. (2006) suggest that most stressful conditions lead to a quick increase in level of oxidatively modified proteins. Thus, OMP levels have risen as a result of other kinds of stressors (Stadtman and Levine 2000). Accumulation of oxidized proteins has also been found during aging and in some disorders (Sohal 2002). ROS can attack multiple cellular constituents, including protein, nucleic acids, and lipids, leading to disruption of cellular function and integrity (Sturve et al. 2008). The formation of oxidative-modified protein causing conformational changes, decreased catalytic activity in enzymes and ultimately resulting, owing to increased susceptibility to protease action, in breakdown of proteins by proteases (Almroth et al. 2008). Our results show that the vaccination against furunculosis caused decrease level of oxidative modification of protein in muscles, gills, and brain of vaccinated fish as compared with the control-unhandled group. On the contrary, level of oxidative modification of protein was higher in liver of vaccinated fish as compared with the other tissues. This is obviously related to the functional activity of liver. The liver plays a key role in most metabolic processes, especially detoxification and, consequently, in the formation of ROS.

ROS in cells may lead to an elevation of antioxidant enzymes as a defense mechanism (Velisek et al. 2011). Antioxidants provide cells with a comprehensive defense from ROS-induced damage. These defenses include low molecular weight compounds (e.g., glutathione and ascorbate) and antioxidant enzymes (e.g., superoxide dismutase, catalase, and glutathione-dependent antioxidant enzymes) (Grim et al. 2013). Glutathione (GSH), a tri-peptide that can neutralize ROS, also serves as a critical cofactor for several glutathione-dependent antioxidant enzymes (Pacitti et al. 2013). The first line of defense against oxidative stress consists of the antioxidant enzymes SOD, CAT, and GPx, which converts superoxide radicals into hydrogen peroxide and then into water and molecular oxygen (Ural 2013). Induction of antioxidant enzymes is an important line of defense against oxidative stress in fish (Velisek et al. 2011). SOD is a group of metalloenzymes that catalyzes the dismutation of superoxide to hydrogen peroxide, plays a crucial antioxidant role and constitutes the primary defense against the toxic effects of superoxide radicals in aerobic organisms (Cheeseman and Slater 1992). In our study, significant increase in SOD activity was observed in muscles and liver of vaccinated trout. It could be adaptive response to the immunization that neutralizes the impact of ROS and may be of importance in preventing membrane lipid peroxidation when the latter is initiated by a combination of Fe^3+^ and O_2_^−•^-generating system (Cadenas et al. 1992). A similar result of increased SOD activity has been reported in carp tissues following xenobiotics exposure (Ural 2013; Oruç 2010).

CAT, associated with other enzymatic antioxidants (peroxidases, SOD), is capable of removing, neutralizing, or scavenging ROS and is, with the GSH redox cycle, the primary cellular enzymatic defense system against hydrogen peroxide, that it converts to H_2_O and O_2_ (Dorval and Hontela 2003). The decreased CAT activities indicate the reduced capacity to scavenge hydrogen peroxide produced in muscles and gills of vaccinated trout in response to oxidative stress. Similarly, the inhibition of the CAT activity by pesticides has been reported in various studies in fish species (Ural 2013). For example, Oruç and Usta (2007) reported that diazinon caused a decrease in the CAT activity in the muscle of *Cyprinus carpio.* Similarly, influence of chlorpyrifos significantly decreased CAT activities in the liver, kidney, and gills of *Cyprinus carpio* (Ural 2013). In our study, the CAT activity was also significantly decreased in the muscles and gills of vaccinated trout. The decrease in the CAT activity observed in the tissues could be due to the production of superoxide radicals under the vaccination. At that time, superoxide radicals can inhibit CAT activity (Kono and Fridovich 1982).

The most important antioxidant enzymes linked with antioxidant defenses against oxidative stress are glutathione peroxidase, reductase, and transferase (Hayes and McLellan 1999). The activity of antioxidant enzymes, such as GPx and other markers of oxidative stress, has been extensively used in fish both in vivo and in vitro studies (Dörr et al. 2008; Misra and Niyogi 2009; Misra et al. 2012).

GR plays an important role in cellular antioxidant protection and adjustment processes of metabolic pathways (Cazenave et al. 2006). It catalyzes the reduction of glutathione disulfide to reduced glutathione in an NADPH-dependent reaction. In our study, GR activity was significantly decreased in the muscles and gills of vaccinated trout, but increased in the liver, probably due to an availability of NADPH in different tissues.

Inactivation of lipid-derived hydroperoxides can be catalyzed by GSH-dependent selenoperoxidases or certain non-seleno-GSH-S-transferases. Two selenoperoxidases are known to exist in cells: classical GSH-peroxidase (GPx), which acts on relatively polar substrates, e.g., H_2_O_2_ or fatty acid hydroperoxides, and phospholipid hydroperoxide GSH-peroxidase (Ursini and Bindoli 1987). GPx is dependent on access to glutathione disulfide by the NADPH-dependent GR. Decrease in glutathione-mediated antioxidant defense system results in oxidative stress and increased cytotoxicity, whereas elevation of intracellular GSH levels is recognized as an adaptive response to oxidative stress (Sagara et al. 1998). In our study, the activities of GPx, as well as GR were significantly decreased in the muscles and gills of vaccinated trout.

Total antioxidant capacity in muscles, liver, gills, and brain of vaccinated trout was significantly decreased when compared with the unhandled group. Impairment in the synthesis of enzymatic and nonenzymatic antioxidant of vaccinated fish may be the most important factor in reducing levels of cellular total antioxidant. Similar result was obtained by Banaee et al. (2013). The results showed that diazinon, which is widely used as insecticide, altered the activity of antioxidant enzymes and decreased the TAC-inducing oxidative stress and cellular damage in hepatocytes of rainbow trout (Banaee et al. 2013).

Our results suggest that both the glutathione-mediated antioxidant defense system and endogenous CAT play a critical role in intracellular antioxidant defense in vaccinated fishes. At the same time, the antioxidant defenses  was significant higher in liver of vaccinated fishes, probably due to a functional activity of liver. The importance of the glutathione-mediated antioxidant defense system in protection against endosulfan-induced oxidative stress was also demonstrated in adrenocortical cells of rainbow trout (Dorval and Hontela 2003).

Oxidative stress biomarkers analyses revealed significant differences between vaccinated fish against furunculosis. We noted strong association between oxidative stress and tissues responses. The liver tissue of vaccinated fish showed higher levels of lipid and protein oxidation biomarkers. Rainbow trout muscle, gills, liver, and brain tissue showed different antioxidant defense responses, likely related to tissue-specific functions. Furthermore, changes in lipid and protein oxidation of vaccinated trout were also tissue-dependent. Muscle, gill, and brain tissues had lower level of aldehyde and ketonic derivates of oxidatively modified protein, while liver tissue became more susceptible to oxidative damage induced by vaccination. Muscle and gill glutathione-dependent enzymes activity decreased in vaccinated trout. In contrast, antioxidant defenses in liver tissue increased, which indicate a different response of tissue to vaccination. Our data suggest that vaccination against furunculosis induced oxidative stress in gill and liver tissues. However, muscle and brain tissue are capable of restoring its pro- and antioxidant balance after vaccination.
